# Single Crystals of EuScCuSe_3_: Synthesis, Experimental and DFT Investigations

**DOI:** 10.3390/ma16041555

**Published:** 2023-02-13

**Authors:** Maxim V. Grigoriev, Anna V. Ruseikina, Vladimir A. Chernyshev, Aleksandr S. Oreshonkov, Alexander A. Garmonov, Maxim S. Molokeev, Ralf J. C. Locke, Andrey V. Elyshev, Thomas Schleid

**Affiliations:** 1 Laboratory of Theory and Optimization of Chemical and Technological Processes, University of Tyumen, Tyumen 625003, Russia; 2 Institute of Inorganic Chemistry, University of Stuttgart, D-70569 Stuttgart, Germany; 3 Institute of Natural Sciences and Mathematics, Ural Federal University named after the First President of Russia B.N. Yeltsin, Mira Str. 19, Ekaterinburg 620002, Russia; 4 Laboratory of Molecular Spectroscopy, Kirensky Institute of Physics, Federal Research Center KSC SB RAS, Krasnoyarsk 660036, Russia; 5 School of Engineering and Construction, Siberian Federal University, Krasnoyarsk 660041, Russia; 6 Institute of Physics and Technology, University of Tyumen, Tyumen 625003, Russia; 7 Institute of Engineering Physics and Radioelectronic of Siberian State University, Krasnoyarsk 660041, Russia; 8 Laboratory of Crystal Physics, Kirensky Institute of Physics, Federal Research Center KSC SB RAS, Krasnoyarsk 660036, Russia; 9 Department of Physics, Far Eastern State Transport University, Khabarovsk 680021, Russia

**Keywords:** quaternary chalcogenides, crystal structure, DFT calculations, semiconductors, vibrational spectroscopy

## Abstract

EuScCuSe_3_ was synthesized from the elements for the first time by the method of cesium-iodide flux. The crystal belongs to the orthorhombic system (*Cmcm*) with the unit cell parameters *a* = 3.9883(3) Å, *b* = 13.2776(9) Å, *c* = 10.1728(7) Å, *V* = 538.70(7) Å^3^. Density functional (DFT) methods were used to study the crystal structure stability of EuScCuSe_3_ in the experimentally obtained *Cmcm* and the previously proposed *Pnma* space groups. It was shown that analysis of elastic properties as Raman and infrared spectroscopy are powerless for this particular task. The instability of EuScCuSe_3_ in space group *Pnma* space group is shown on the basis of phonon dispersion curve simulation. The EuScCuSe_3_ can be assigned to indirect wide-band gap semiconductors. It exhibits the properties of a soft ferromagnet at temperatures below 2 K.

## 1. Introduction

Copper-containing chalcogenides have recently attracted considerable interest due to their promising thermoelectric applications [1,2], owing to their low thermal conductivity [2,3]. The compounds can be used in solar cells [4], photocatalysts [5], and gas sensing [6,7].

The chemistry of trivalent scandium compounds is of particular interest in terms of crystal structure and properties [8]. Trivalent scandium differs from other trivalent cations of the first transition series due to the presence of a closed outer electron shell with an argon configuration. Scandium chalcogenides are *p*-type [2,4,9,10,11] and *n*-type [8] semiconductors. Doping with heavy metals results in *n*-type conduction with low resistivity [9] in compounds that exhibit metallic properties [12].

The band gap of scandium chalcogenides compounds varies over a wide range from 1.2–2.3 eV [6,7,13,14].

Scandium chalcogenides can be obtained in different ways:

- In the form of single crystals by the methods of reactive flux or halide flux [15],
- By chemical transport reaction with I_2_ [9],
- High-temperature alloying of elements with chalcogenides [12],
- From the elements by the transport method at 1270 K,
- By the interaction of binary chalcogenides at 1420 K [16],
- As polycrystals by alloying the elements at 1420 K followed by annealing at 870 K for 240 h [17],
- By sulfiding mixtures of oxides obtained by thermolysis of co-crystallized metal nitrates at 870–1170 K for 25 h [13].

For ternary scandium-copper chalcogenides ScCu*Ch*_2_, their structural stability, thermal lattice conductivity, transport, and thermoelectric properties were appreciated [2]. ScCuSe_2_ has the highest Q factor of 0.65 at 1000 K among the chalcogenides. This makes it a potential candidate for high-temperature thermoelectric applications. [2]. Down the group of chalcogenides ScCu*Ch*_2_ (*Ch* = S, Se, and Te), there is a decrease in the elastic moduli and values of the Debye temperatures, the gap width [2,18]. Ternary compounds ScCu*Ch*_2_ are semiconductors with an indirect gap, with a minimum of the conduction band at the high symmetry L point and a maximum of the valence band at the Γ point [18]. ScCu*Ch*_2_ is considered for copper-based n-window applications [2].

Quaternary scandium chalcogenides exhibit a variety of magnetic properties. EuScCuS_3_ shows a ferromagnetic transition at 3–9 K [13,14], SrScCuS_3_ diamagnetic [13], and antiferromagnetic [14]. The replacement of Sr^2+^ by Eu^2+^ leads to a narrowing of the band gap due to the 4*f*–5*d* transition in the Eu^2+^ cations. This makes it possible to control the band gap of the chalcogenides by including europium. The activation energy of defects in the crystal structure, which is a source of additional absorption in the NIR spectral range, turned out to be 0.29 eV [13]. The EuScCuSe_3_ compound should treat materials with excellent *p*-type semiconductor conductivity. The upper part of the valence band consists of Cu *3d* states overlapping with S *3p* states similar to Cu_2_S [19] and *A*Cu*Ch*_2_ materials [4,20].

The structure type of NaCuTiS_3_ was predicted for EuScCuSe_3_, and quantum mechanical calculations were performed using the PBE functional, according to which the band gap of this selenide was 0.82 eV [3]. The NaCuTiS_3_ compound crystallizes in the orthorhombic system (space group Pnma) with the lattice parameters *a* = 12.738 Å, *b* = 3.554 Å, and *c* = 9.529 Å [21]. The structure is represented by layers 2D-[CuTiS_3_]^–^ consisting of alternating pairs of distorted tetrahedra [CuS_4_]^7–^ and octahedra [TiS_6_]^8–^ in the direction [001], which are separated by single-capped trigonal prisms [NaS_7_]^13–^ [21]. However, previously synthesized quaternary scandium chalcogenides EuScCuS_3_ [11], SrScCuS_3_ [11,22], SrScCuSe_3_ [1,23], BaScCuS_3_ [24] crystallize in the space group *Cmcm* with structure type of KZrCuS_3_. Since the ionic radius of Eu^2+^ (*r_i_*(Eu^2+^) = 1.17 Å, *C.N.* = 6 [25]) is close to the ionic radius of Sr^2+^ (*r_i_(*Sr^2+^) = 1.18 Å, *C.N.* = 6 [25]), it is expected that the EuScCuSe_3_ compound will crystallize in the same structure type as and SrScCuSe_3_ [1,23].

There are no data in the literature on the synthesis, crystal structure, or any properties of EuScCuSe_3_. In this work, we describe the synthesis of EuScCuSe_3_ for the first time, the studies of its crystal structure, as experimentally as well as using theoretical methods, and the investigation of its magnetic properties. Simultaneously, taking into account that most of the photonic media are single crystals, it has been decided to synthesize EuScCuSe_3_ in that form.

## 2. Materials and Methods

### 2.1. Materials and Synthesis

**Figure 1:**
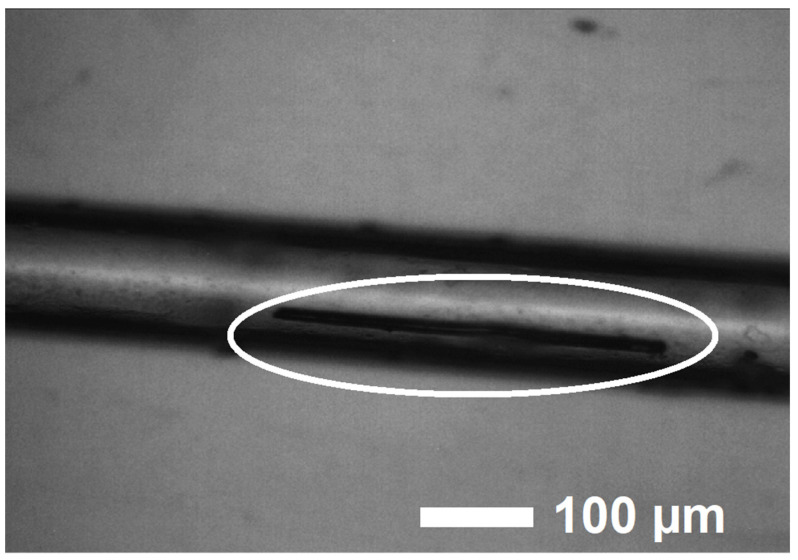
A single crystal of EuScCuSe_3_ placed in a glass capillary.

The following chemical reagents were used: Eu (99.3%), Sc (99.9%), CsI (99.9%), Se (99.9%) were procured from ChemPur (Karlsruhe, Germany), and Cu (99.999%) stemmed from Aldrich (Milwaukee, WI, USA). EuScCuSe_3_ was synthesized by the halide-flux method. The work was carried out in a glove box under an argon gas inert atmosphere. The stoichiometric ratio of the elements of europium (76.39 mg), scandium (22.60 mg), copper (31.94 mg), and selenium (119.07 mg) in the presence of CsI (800 mg) was loaded into silica ampoules. These ampoules were evacuated to a pressure of 2 × 10^−3^ mbar, sealed, and then heated in a resistant furnace. A temperature of 1120 K was reached within 30 h and kept for 96 h. Afterward, it was cooled to 570 K at a rate of 4 K h^−1^ and then to room temperature within 3 h. The reaction proceeded according to the equation: Eu + Cu + Sc + 3 Se → EuScCuSe_3_. The reaction product was purified from flux residues with demineralized water. The synthesized samples were dark red, needle-shaped single crystals of EuScCuSe_3_ (Figure 1).

### 2.2. Methods

A selected single crystal of EuScCuSe_3_ 0.05 × 0.05 × 0.45 mm^3^ in size was sealed into a thin-walled glass capillary (Figure 1) for X-ray diffraction experiments. The capillary was subsequently mounted on a Bruker-Nonius κ-CCD single-crystal diffractometer (Bruker, Billerica, MA, USA) equipped with a Mo-*K*_α_ radiation source, a graphite monochromator, and a CCD detector. The unit cell of this compound belongs to the orthorhombic crystal system. The space group was determined from a statistical analysis of the intensities of all reflections. The DENZO program [26] was used to process the collected intensity data. The HABITUS program [27] was used to numerically correct the absorption. The crystal structure was solved and refined by means of the SHELX-2013 software package [28,29]. CSD 2,239,558 contains supplementary crystallographic data. These data can be obtained free of charge via https://www.ccdc.cam.ac.uk/structures (accessed on 11 February 2023) or from the Cambridge Crystallographic Data Centre, 12 Union Road, Cambridge CB2 1EZ, UK; fax: (+44)1223-336-033; or e-mail: deposit@ccdc.cam.ac.uk. Crystal structures were visualized in the program package VESTA 3.5.7 [30].

The temperature dependence of the EuScCuSe_3_ magnetization was measured using a helium-cooled magnetic property measurement system (MPMS3, Quantum Design, San Diego, CA, USA) in the temperature range from 2 to 300 K in zero-field cooling (ZFC) modus and heating in an external magnetic field (FC). The field value was 500 kOe (39.8 mA m^−1^). The field-dependent magnetic moments were measured at room temperature (300 K) and at 2 K.

The ab-initio calculations of the EuScCuSe_3_ were carried out in the framework of density functional theory (DFT) using the PBE0 exchange-correlation functional [18], which takes into account both local and nonlocal *Hartree*–*Fock* exchanges. The calculations were performed in the CRYSTAL17 program designed to simulate periodic structures [31,32]. For Eu^2+^, the ECP*53*MWB quasi-relativistic pseudopotential was used to describe the inner shells of this lanthanoid cation. Thus, the inner shells, including 4, were replaced by a pseudopotential. To describe the outer shells (5*s*^2^5*p*^6^) involved in chemical bonds, a valence basis set of TZVP type was used. The pseudopotential and the valence basis set are available on the site [4].

For scandium, copper, and selenium, the full-electron basis sets were used. The basis sets are available on the CRYSTAL program site as «Sc_864–11G*_harrison_2006», «Cu_86–4111(41D)G_doll_2000» and «Se_976–311d51G_towler_1995» [32]. *Gaussian* primitives with orbital exponent values less than 0.1 were removed from the basis sets since these calculations are periodic. The exponent in the outer orbital of selenium was set to 0.14. The accuracy of calculating the self-consistent field was set to 10^−9^ a.u. The accuracy of the calculation of the two-electron integrals was set to at least 10^−8^. Integration over the *Brillouin* zone was carried out according to the *Monkhorst-Pack* scheme with a grid of k-points equal to 8 × 8 × 8. The sequence of calculations was as follows. The optimization of the crystal structure was carried out first. After that, the phonon spectrum was calculated at the Г point, or the elastic constants were calculated for the crystal structure corresponding to the minimum energy.

## 3. Results and Discussion

### 3.1. Crystal Structure

The crystal structure was determined from single-crystal X-ray diffraction data. Crystallographic and structural data are described in Table 1 and Table 2 as well as Tables S1–S3 of the Supplementary Materials (SM).

**Figure 2:**
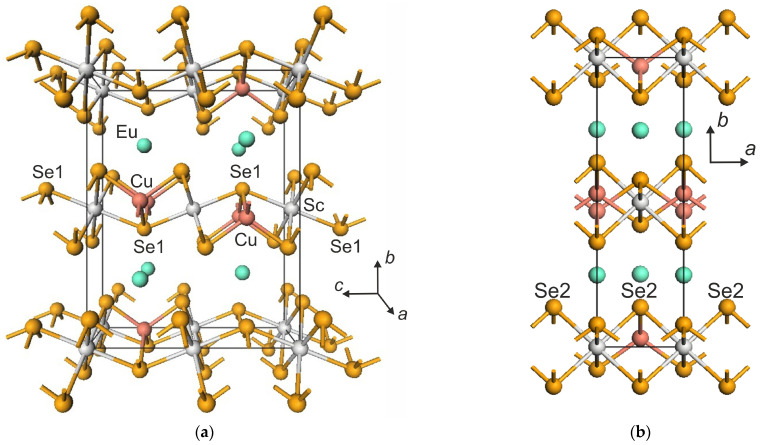
Orthorhombic crystal structure of EuScCuSe_3_: (**a**) Perspective view of the extended unit cell perpendicular onto the *bc* plane, (**b**) projection onto the *ab* plane.

The octahedral [ScSe_6_]^9−^ units in the EuScCuSe_3_ structure are interconnected to each other through the (Se1)^2−^ ions along the *z* axis, as shown in Figure 2a, and though the (Se2)^2−^ anions along *a* axis (see Figure 2b). The [CuSe_4_]^7−^ tetrahedra are linked via common (Se1)^2−^ anions along *a* axis. The [ScSe_6_]^9−^ and [CuSe_4_]^7−^ units have common Se1 and Se2 vertices. The nearest neighbors around Eu^2+^ cations form trigonal prisms [EuSe_6_]^10−^ (Figure S1). The four Eu–Se1 bond lengths are equal to 3.0605 Å, while the remaining two Eu–Se2 bonds are 3.1711 Å long (Table S2).

### 3.2. Density Functional Theory Calculations

As has been mentioned above, a crystal structure prediction was previously made for EuScCuSe_3_, and the space group *Pnma* was supposed [3]. Due to the fact that the sample experimentally synthesized in our work was solved in space group *Cmcm*, we did a comprehensive investigation of the EuScCuSe_3_ crystal structure stability in both space groups, *Pnma* and *Cmcm*.

At the first step of density functional theory calculations, crystal structures of EuScCuSe_3_ in *Pnma* and *Cmcm* space groups were totally optimized, and the obtained lattice parameters are presented in Table 3. The simulated structural data get close to the experiments in both cases. It should be noted that the energy per formula unit is almost the same for both structure types and differs only in the fifth decimal place: −9634.327521165 at. un (*Pnma*), −9634.327541515 at. un (*Cmcm*).

The next mandatory part of the crystal structure stability investigation is the simulation of elastic properties [33]. Calculations of the elastic constants were performed using the built-in functionality of CRYSTAL17 code. The obtained data for EuScCuSe_3_ in *Cmcm* and *Pnma* structures are presented in Table 4. The necessary and sufficient *Born* criteria [34] for the orthorhombic crystal-system stability are *C*_11_ > 0, *C*_11_*C*_22_ > *C*_12_, *C*_11_*C*_22_*C*_33_ + 2*C*_12_*C*_13_*C*_23_ − C_11_C_23_^2^ − C_22_C_13_^2^ − C_33_C_12_^2^ > 0, C_44_ > 0, C_55_ > 0, C_66_ > 0. All the above conditions are satisfied both for the real *Cmcm* and predicted *Pnma*-structure of EuScCuSe3 previously.

As any data on elastic properties of EuScCuSe_3_ are absent at this time in databases or articles, we present calculation of the bulk modulus, *Young*’s modulus, and shear modulus in the *Voigt*, *Reuss*, and *Hill* approximations (Table 5). The dependence of *Young*’s modulus on the crystal directions demonstrates a significant anisotropy of the elastic properties in both the *Cmcm* and the *Pnma* structure (Figure S2).

The calculated values of the shear modulus and bulk modulus make it possible to estimate the *Vickers* hardness for EuScCuSe_3_ (Table 5). To estimate the *Vickers* hardness, the empirical formula (3.3.1) from work [1] was used.

*H_v_* = 0.92(*G*/*B*)^1.137^*G*^0.708^ (1)

This formula well describes the hardness of a row of compounds with an ionic and covalent type of chemical bond (about 40 compounds were considered in work [1]). In Formula (1), *G* and *B* are the shear modulus, and bulk modulus by *Hill* is estimated. The experimental values of hardness are absent from research papers. According to calculations, the elastic constants and hardness of EuScCuSe_3_ differ significantly for the *Cmcm* and *Pnma* structures (Table 4).

As vibrational spectroscopy is a powerful tool for the determination of crystal structure details, simulation of Raman and infrared spectra for the experimentally obtained data in this work (*Cmcm* structure) and possibly earlier predicted *Pnma* structure [1] were done. The results for the infrared-active modes, Raman modes, and “silent” modes at the Г point are given in Tables S4 and S5 of the SM. The degree of participation of each ion in a particular mode is estimated from the analysis of displacement vectors obtained from these ab-initio calculations. The ions that are shifted significantly in the mode are listed in the column “participants” (Tables S4 and S5). The values of ion displacements for vibrational modes are shown in Figure S3.

**Figure 3:**
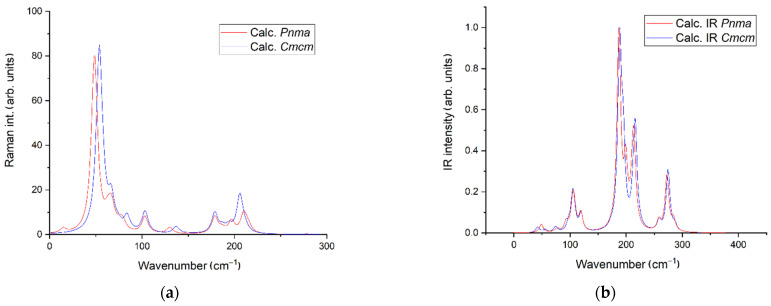
Simulated Raman (**a**) and infrared spectra (**b**) for EuScCuSe_3_ in its two possible structures. All calculations were performed for *T* = 298 K.

The number of formula units in the *Pnma* structure is equal to 4 (Z = 4), and this value is the same for the *Cmcm* structure, see Table 1. However, the primitive cell of the *Cmcm* structure contains only two formula units (Figure S4). Thus, the number of vibrational modes should be larger in the *Pnma* case. The Raman-active modes for *Pnma* and *Cmcm* structures should be listed as 12 *A_g_* + 6 *B*_1*g*_ +12 *B*_2*g*_ + 6 *B*_3*g*_ and 5 *A_g_* + 4 *B*_1*g*_ + *B*_2*g*_ + 5 *B*_3*g*_, correspondingly [35]. The result of Raman and infrared spectra simulations for both structures are presented in Figure 3. Despite the fact that the number of vibrational modes is different for the structures in *Cmcm* and *Pnma*, the simulated Raman and infrared spectra are quite similar. Thus, we suppose that the definition of the correct space group (*Cmcm* or *Pnma*) using experimental vibrational spectroscopy is almost impossible in this case.

**Figure 4:**
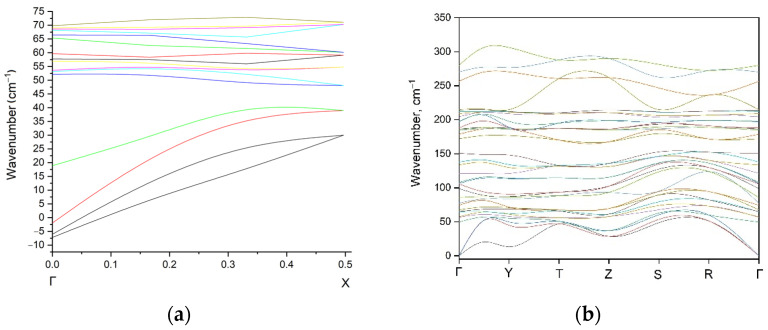
Calculated phonon dispersion curves of EuScCuSe_3_ in (**a**) *Pnma* and (**b**) *Cmcm* structures.

The only possible indicator for the *Pnma* structure is the low-lying weak band in the Raman spectrum (Figure 3a) which is associated with very strong movements of all ions except for Cu^+^ (Figure S3). However, the wavenumber value of this vibrational mode is the lowest in both structures. In this regard, the calculation of phonon dispersion curves was done for the *Pnma* structure, and the results of the simulation in Γ–X direction are shown in Figure 4a. The key factor of the dynamical stability of crystal lattice is the absence of imaginary (unstable) phonon modes and this approach works in for the case of experimentally observed crystal structures [36] as for crystal structure stability prediction [37,38]. According to the obtained data (Figure 4a), we can say that the crystal structure of EuScCuSe_3_ in the previously supposed space group *Pnma* should be unstable. This fact, among other things, is consistent with the experimentally obtained space group *Cmcm* obtained for the real EuScCuSe_3_ in this work. At the same time, simulated phonon dispersion for the *Cmcm* structure do not contain unstable phonon modes over all of the high-symmetric *Brillouin* zone points (*Cmcm*).

**Figure 5:**
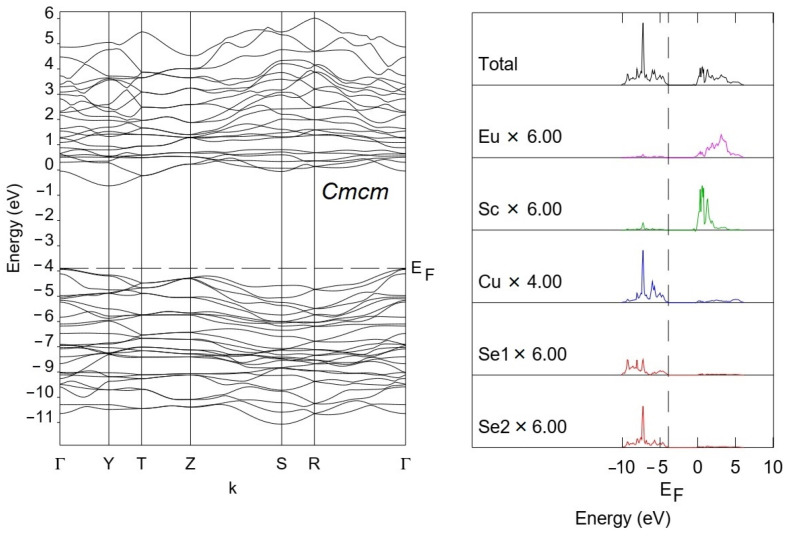
Band structure and electronic density of states for EuScCuSe_3_.

The band structure and the density of states for EuScCuSe_3_ calculated using hybrid PBE0 functional are shown in Figure 5. The path in the *Brillouin* zone is plotted through the most highly symmetric points. For the space group *Cmcm*, the path is made along Г–Y–T–Z–S–R–Г. The coordinates of the points are (0,0,0,), (^1^/_2_,^1^/_2_,0), (^1^/_2_,^1^/_2_,^1^/_2_), (0,0,^1^/_2_), (0,^1^/_2_,0), (0,^1^/_2_,^1^/_2_), (0,0,0) respectively. The Bilbao crystallographic server was used [35]. Since for europium pseudopotential that replaced their core shells, the 4*f* inclusive was used, the band structure does not include 4*f* states. For the Eu^2+^ cations, only outer shells (5*s*^2^5*p*^6^) were taken into account by means of valence basis sets [39]. The projected DOS onto the whole set of atomic orbitals of Eu, Sc, Cu, and Se atoms was calculated near the band gap. According to these calculations, the DOS of copper and selenium are located near the top of the valence band. The DOS of scandium and europium are located near the bottom of the conduction band. The band gap value is defined as the difference in energy between the top of the valence band and the bottom of the conduction band. Calculations predict for EuScCuSe_3_ the indirect electronic transition with a band gap value of 3.27 eV. It should be noted, that in the case of the dynamically unstable *Pnma* structure, the band gap value is the same, but the calculated electronic transition is direct (Figure S5).

### 3.3. Magnetic Properties

**Figure 6:**
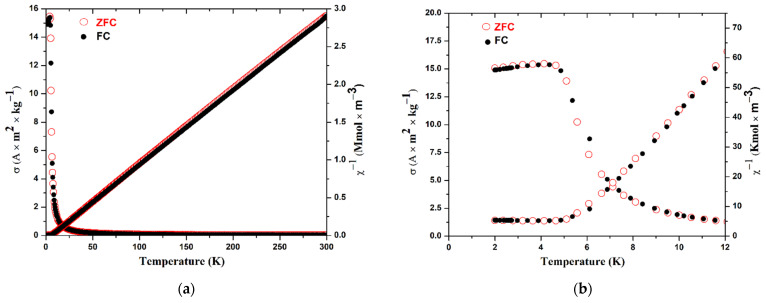
Temperature dependences of specific magnetization *σ* (left axis) and reciprocal molar susceptibility *χ*^−1^ (right axis) in the temperature range from 2 K to 300 K (**a**) and to 12 K (**b**).

The temperature dependence of the specific magnetization was measured in the temperature range from 2 to 300 K (Figure 6). Based on it, the temperature dependences of the direct and reciprocal values of the molar magnetic susceptibility are calculated.

The main contribution to the magnetic properties of EuScCuSe_3_ is made by the Eu^2+^ cations with unfilled *f-*shells. There is no significant effect of the crystal field on the magnetic moment since, in the ground state (^8^S_7/2_), this cation has a zero-orbital momentum. Its temperature dependence of magnetic susceptibility in the paramagnetic region should be well described by the *Curie-Weiss* law: $\chi =\chi_{\mathrm{TIP}}+\frac{C}{T-\theta_{W}}$ considering the temperature-independent term χ_TIP_. Approximation of the experimental dependence by this formula gives the following values: χ_TIP_ = 1.04·10^−5^ m^3^ kmol^−1^, *C* = 0.0977 K m^3^ kmol^−1^, *θ*_W_ = 6.0 K. The deviations of the experimental points from the approximating curve in the temperature ranging from 40 to 300 K are no more than 1%, and from 10 to 40 K about 2.5%. A comparison of the characteristics obtained with those calculated for non-interacting Eu^2+^ cations is given in Table 6.

There is a sharp deviation from the *Curie-Weiss* law at temperatures below 5 K. This deviation is obviously due to the ferromagnetic transition, although there is no noticeable discrepancy in the data for the FC and ZFC

**Figure 7:**
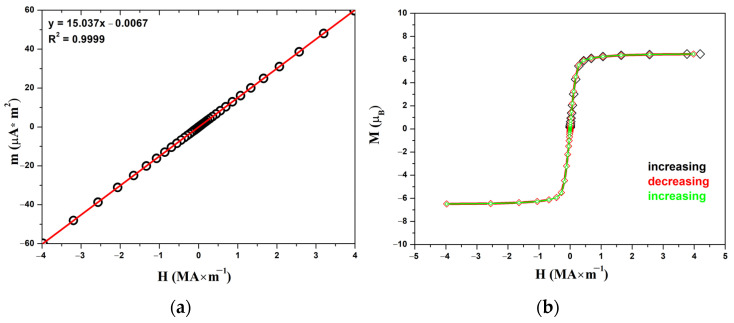
Field-dependent magnetic moments for EuScCuSe_3_ at 300 K (**a**) and magnetization curve at 2 K (**b**).

The experimental curve of magnetization at a temperature of 2 K (Figure 7b) has the form characteristic of magnetically soft ferromagnets. The coercive force is less than 2 kA m^−1^, and saturation occurs in a field of about 500 kA m^−1^. The magnetization in a field of 4 MA m^−1^ per formula unit is 6.5 *μ*_B_, which is close to the theoretical value of about 7 *μ*_B_ for a free Eu^2+^ cation.

## 4. Conclusions

In summary, we report on the new quaternary scandium selenide EuScCuSe_3_, which was synthesized from a mixture of the elements with CsI as a flux in sealed silica ampoules at elevated temperatures. The structural, vibrational, and elastic-property calculations have been performed for EuScCuSe_3_ in the framework of the density functional theory (DFT) by using the PBE0 hybrid functional and LCAO-MO approach. The calculation results predict the *Cmcm* structure, which agrees very well with the obtained crystallographic data (*a* = 3.9883(3), *b* = 13.2776(9), *c* = 10.1728(7) Å). The calculation results can be used to interpret the Raman and infrared spectra.

The crystal structure, according to single-crystal data, showed that EuScCuSe_3_ belongs to the orthorhombic crystal system with the space group *Cmcm*. The structure type corresponds to KZrCuS_3,_ and thus the structure includes trigonal prisms [EuSe_6_]^10−^, octahedra [ScSe_6_]^9−^, and tetrahedra [CuSe_4_]^7−^. The title compound is paramagnetic above 4.5 K and soft ferromagnetic at lower temperatures.

## Figures and Tables

**Table 1:** Crystallographic data for EuScCuSe_3_ and their determination.

Compound	EuScCuSe_3_
Space group	*Cmcm* (no. 63)
Structure type	KZrCuS_3_
*a* (Å)	3.9883 (3)
*b* (Å)	13.2776 (9)
*c* (Å)	10.1728 (7)
*Z*	4
*ρ*_cal_ (g cm^−3^)	6.132
*V*_u_._c_. (Å^3^)	538.70 (7)
Measuring range, ±*h*/±*k*/±*l*	±5/±16/±13
*F*(000)	860
Absorption coefficient *μ* (mm^−1^)	36.73
Measured reflections	5112
Symmetry-independent reflections	367
*R*_int_/*R*_σ_	0.089/0.041
*R*_1_ for *n* reflections with ∣F_o_∣ ≥ 4*σ*(F_o_)	0.055
*n*	294
*R*_1_/*wR*_2_ for all reflections	0.042/0.091
GooF	1.087
Extinction coefficient, ε	0.0016 (4)
Residual electron density, *ρ*_max/min_ (e^−^ 10^6^ pm^−3^)	2.318/−2.273
CSD-number	2239558

**Table 2:** Fractional atomic coordinates of EuScCuSe_3_.

Atom	Site	Symmetry	*x*/*a*	*y*/*b*	*z*/*c*	*U*_eq_ (Å^2^)
Eu	4*c*	*m*2*m*	0	0.75168 (9)	^1^/_4_	0.0232 (5)
Sc	4*a*	2/*m*..	0	0	0	0.0147 (7)
Cu	4*c*	*m*2*m*	0	0.4697 (2)	^1^/_4_	0.0242 (7)
Se1	4*c*	*m*2*m*	0	0.07683 (17)	^1^/_4_	0.0191 (6)
Se2	8*f*	*m*..	0	0.36312 (11)	0.05610 (16)	0.0199 (5)

**Table 3:** Lattice parameters of EuScCuSe_3_ obtained using PBE0 calculations.

Compound		Space Group	Structure Type	Lattice Constants (Å)			*V* (Å^3^)	*ρ* (g cm^−3^)
EuScCuSe_3_	*Calc.*	*Pnma*	Eu_2_CuS_3_	4.01674	13.38926	10.03507	539.698	6.160
EuScCuSe_3_	*Calc.*	*Cmcm*	KZrCuS_3_	4.02028	13.38869	10.02436	539.574	6.162
EuScCuSe_3_	*Exp.*	*Cmcm*	KZrCuS_3_	3.9883(3)	13.2776(9)	10.1728(7)	538.70(7)	6.132

**Table 4:** Elastic constants (GPa) for EuScCuSe_3_.

Compound	Space Group	Structure Type	C_11_	C_12_	C_13_	C_22_	C_23_	C_33_	C_44_	C_55_	C_66_	B	*H_Vcal_*
EuScCuSe_3_	*Pnma*	Eu_2_CuS_3_	122	33	38	150	44	99	46	21	33	66	5.7
EuScCuSe_3_	*Cmcm*	KZrCuS_3_	151	43	27	98	37	128	13	33	45	65	4.8

**Table 5:** Bulk (*B*), shear (*G*), and *Young*’s modulus (GPa) of EuScCuSe_3_.

Compound	Space Group	Structure Type	Averaging Scheme	*B*	*G*	*Young*’s Modulus	*Poisson* Ratio
EuScCuSe_3_	*Pnma*	Eu_2_CuS_3_	*Voigt*	67	37	94	0.263
			*Reuss*	65	34	86	0.278
			*Hill*	66	36	90	0.271
EuScCuSe_3_	*Cmcm*	KZrCuS_3_	*Voigt*	66	36	92	0.267
			*Reuss*	64	28	74	0.309
			*Hill*	65	32	82	0.287

**Table 6:** Magnetic characteristics for EuScCuSe_3_.

Magnetic Characteristics	Calculated	M(H) at 300 K	M(T) at 500 kOe
*C* (K m^3^ kmol^−1^)	0.098999	0.0995	0.0977
*μ* (μ_B_)	7.9373	7.96	7.89
*θ*_W_ (K)	-	-	6.0
*T*c (K)	-	-	4.5

## Data Availability

Data are available from the authors on request.

## Supplementary Materials

The following supporting information can be downloaded at: https://www.mdpi.com/article/10.3390/ma16041555/s1, Figure S1: Crystal structure of EuScCuSe_3_. Projection onto the *bc* plane (a) and onto the *ab* plane (b). The trigonal prisms [EuSe_6_]^10−^ are colored in turquoise; Figure S2: Dependence of *Young*’s modulus in GPa on the crystallographic directions in EuScCuSe_3_ for both possible orthorhombic structures; Figure S3: Displacement of ions at the phonon modes in the crystal structure of EuScCuSe_3_ in both possible descriptions (*Cmcm* and *Pnma*); Figure S4: Primitive cell of EuScCuSe_3_; Figure S5: Band structure and electronic density of states of EuScCuSe_3_ calculated for the dynamically unstable *Pnma* structure; Table S1: Anisotropic displacement parameters in Å^2^ of EuScCuSe_3_; Table S2: Main bond lengths in Å of EuScCuSe_3_; Table S3: Geometric parameters for EuScCuSe_3_; Table S4: Wavenumbers in cm^−1^ and types of the phonon modes at the Г-point for EuScCuSe_3_ in the *Cmcm* structure. The intensity of the Raman modes was calculated for *λ* = 532 nm and *T* = 298 K; Table S5: Wavenumbers in cm^−1^ and types of the phonon modes at the Г-point for EuScCuSe_3_ in the *Pnma* structure. The intensity of the Raman modes was calculated for *λ* = 532 nm and *T* = 298 K.
