# Supplementary material for: Single Crystals of EuScCuSe3: Synthesis, Experimental and DFT Investigations
Source: Materials (Basel). 2023 Feb 13;16(4):1555. doi: 10.3390/ma16041555 (PMC9962736; doi:10.3390/ma16041555)
Supplement: Supplementary file 1 [file materials-16-01555-s001.zip › materials-2195111-supplementary.pdf]

materials

Single Crystals of $EuScCuSe_3$: Synthesis, Experimental and DFT Investigations

Maxim V. Grigoriev [1,2,*], Anna V. Ruseikina [1], Vladimir A. Chernyshev [3], Aleksandr S. Oreshonkov [4,5,*], Alexander A. Garmonov [6], Maxim S. Molokeev [7,8,9], Ralf J. C. Locke [2], Andrey V. Elyshev [1] and Thomas Schleid [2,*]

[1] Laboratory of Theory and Optimization of Chemical and Technological Processes, University of Tyumen, 625003 Tyumen, Russia
[2] Institute of Inorganic Chemistry, University of Stuttgart, D-70569 Stuttgart, Germany
[3] Institute of Natural Sciences and Mathematics, Ural Federal University named after the First President of Russia B.N. Yeltsin, Mira Str. 19, 620002 Ekaterinburg, Russia
[4] Laboratory of Molecular Spectroscopy, Kirensky Institute of Physics, Federal Research Center KSC SB RAS, Krasnoyarsk 660036, Russia
[5] School of Engineering and Construction, Siberian Federal University, Krasnoyarsk 660041, Russia
[6] Institute of Physics and Technology, University of Tyumen, Tyumen 625003, Russia
[7] Institute of Engineering Physics and Radioelectronic of Siberian State University, 660041 Krasnoyarsk, Russia
[8] Laboratory of Crystal Physics, Kirensky Institute of Physics, Federal Research Center KSC SB RAS, Krasnoyarsk 660036, Russia
[9] Department of Physics, Far Eastern State Transport University, Khabarovsk 680021, Russia
* Correspondence: ma.v.grigorev@utmn.ru (M.V.G.); oreshonkov@iph.krasn.ru (A.S.O.); schleid@iac.uni-stuttgart.de (T.S.)

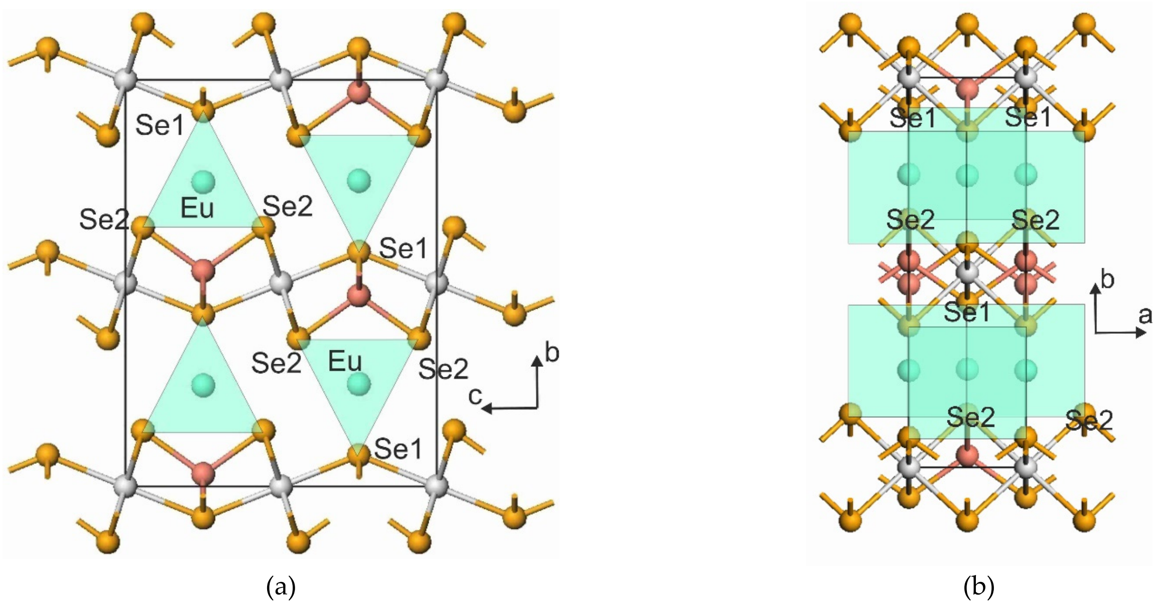

Figure S1. Crystal structure of $EuScCuSe_3$. Projection onto the *bc* plane (a) and onto the *ab* plane (b). The trigonal prisms $[EuSe_6]^{10-}$ are colored in turquoise.

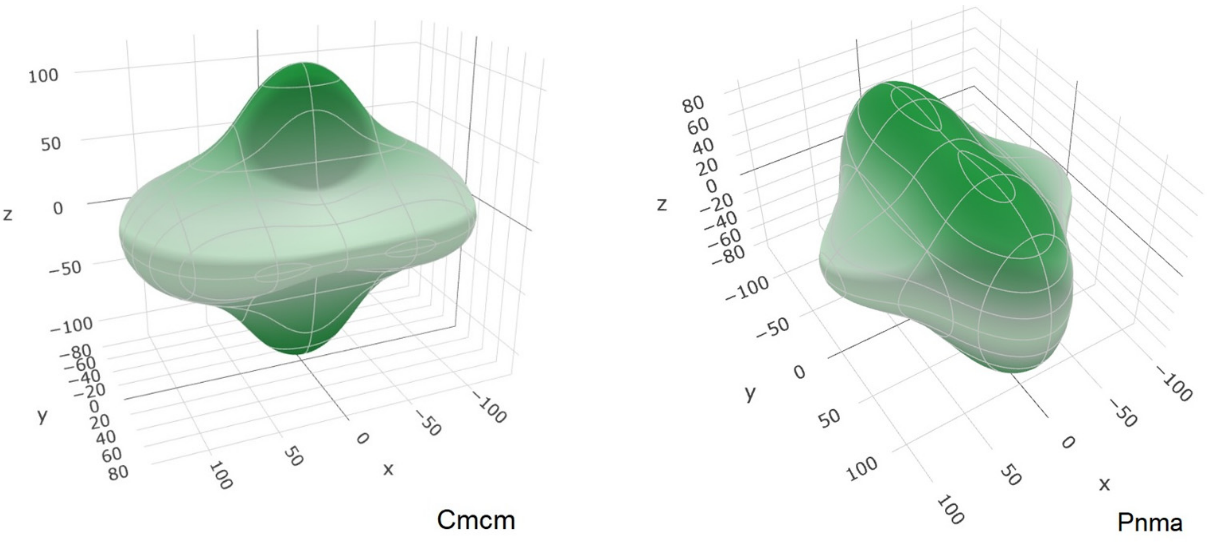

Figure S2. Dependence of the *Young*'s modulus in GPa on the crystallographic directions in EuSc-CuSe3 for both possible orthorhombic structures.

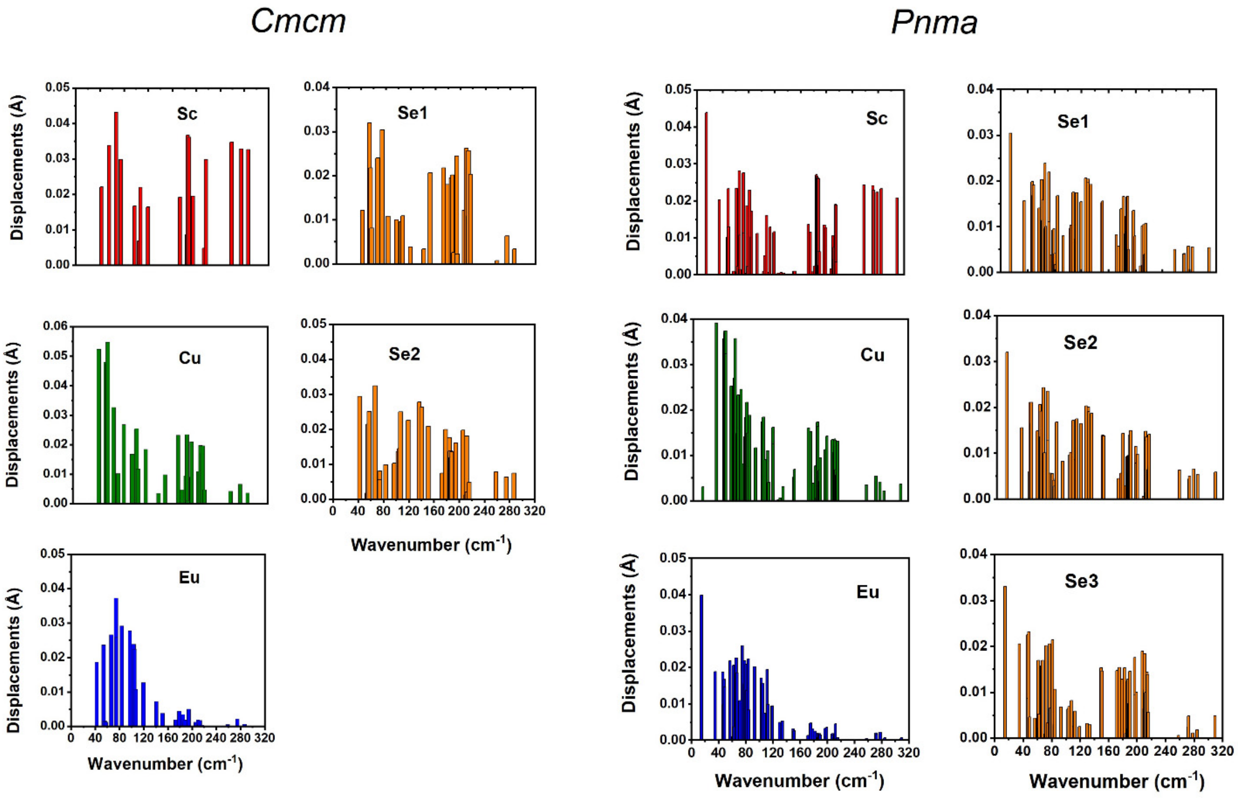

Figure S3. Displacement of ions at the phonon modes in the crystal structure of EuScCuSe3 in both possible descriptions (*Cmcm* and *Pnma*).

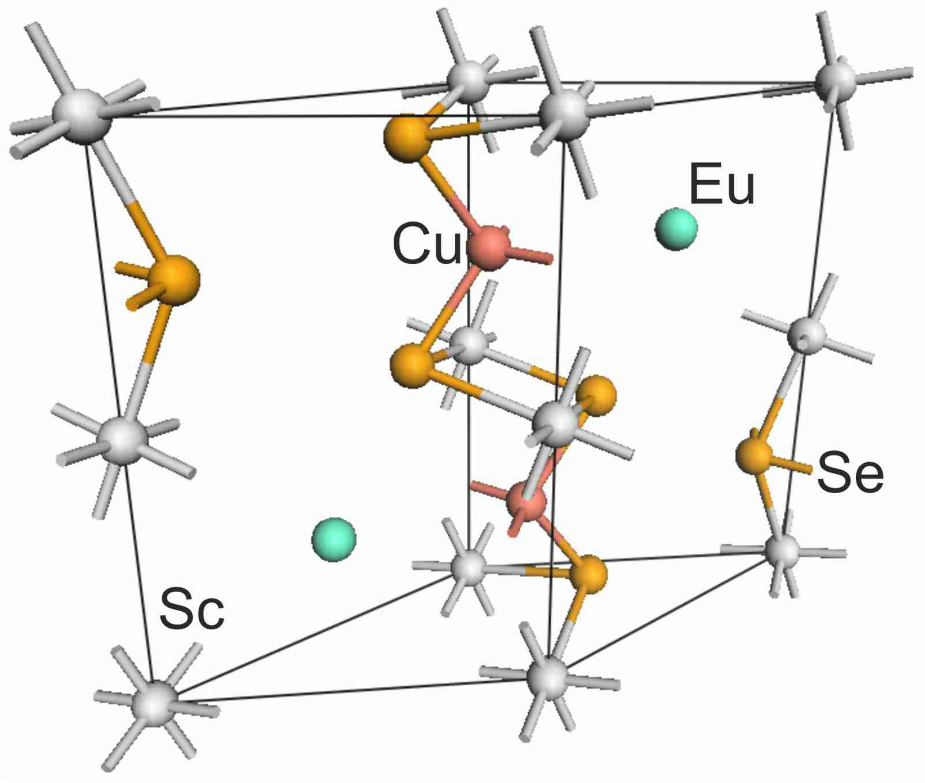

Figure S4. Primitive cell of $EuScCuSe_3$.

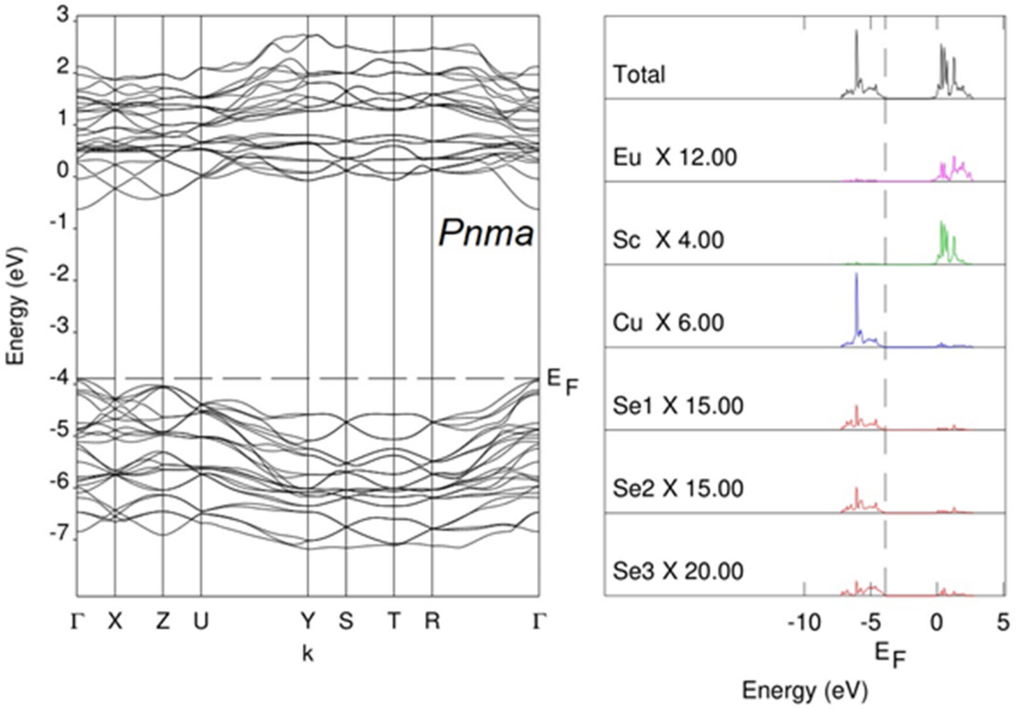

Figure S5. Band structure and electronic density of states of $EuScCuSe_3$ calculated for the dynamically unstable *Pnma* structure.

Table S1. Anisotropic displacement parameters in Å² of $EuScCuSe_3$.

Atom	U_{11}	U_{22}	U_{33}	U_{12}	U_{13}	U_{23}
Eu	0.0164(6)	0.0213(7)	0.0320(8)	0	0	0
Sc	0.0137(16)	0.0143(15)	0.0159(17)	0	0	–0.0002(13)
Cu	0.0230(15)	0.0255(16)	0.0241(16)	0	0	0
Se1	0.0152(11)	0.0203(11)	0.0219(12)	0	0	0
Se2	0.0185(8)	0.0190(9)	0.0222(10)	0	0	–0.0016(6)

Table S2. Main bond lengths in Å of $EuScCuSe_3$.

Eu—Se1^{ii}	2×3.0605(19)	Sc—Se1^{i}	2×2.7401(8)	Cu—Se1^{ii}	2×2.449(2)
Eu—Se2^{ii}	4×3.1711(14)	Sc—Se2^{i}	4×2.7579(10)	Cu—Se2	2×2.428(2)
<Eu—Se>	3.1342	<Sc—Se>	2.7520	<Cu—Se>	2.4385

Symmetry codes $EuScCuSe_3$: (i) –0.5+x, –0.5+y, z; (ii) –0.5+x, 0.5+y, z; (iii) 0.5+x, –0.5+y, z; (iv) –0.5+x, 0.5–y, –z; (v) –x, –y, –0.5+z; (vi) 0.5+x, 0.5+y, z; (vii) –0.5–x, 0.5+y, 0.5–z; (viii) 0.5–x, 0.5+y, 0.5–z; (ix) –x, y, 0.5–z.

Table S3. Geometric parameters for $EuScCuSe_3$.

Atoms 1,2,3	Angle 1,2,3 /°	Atoms 1,2,3	Angle 1,2,3 /°
Se2^{iii}—Sc—Se2^{i}	92.62(4)	Se1—Eu—Se2^{ix}	86.80(3)
Se2^{iv}—Sc—Se2^{i}	87.38(4)	Se1—Eu—Se2	139.79(3)
Se1^{v}—Sc—Se2^{iv}	93.05(5)	Se1—Eu—Se1	81.32(6)
Se1^{v}—Sc—Se2^{iii}	86.95(5)	Se2—Cu—Se2^{ix}	108.68(12)
Se2^{ix}—Eu—Se2	76.92(5)	Se1^{ii}—Cu—Se2	109.79(3)
Se2^{ix}—Eu—Se2^{ix}	77.93(4)	Se1^{ii}—Cu—Se1^{vi}	109.01(14)
Se2^{ix}—Eu—Se2	124.38(6)		

Table S4. Wavenumbers in cm⁻¹ and types of the phonon modes at the Γ-point for $EuScCuSe_3$ in the *Cmcm* structure. The intensity of the Raman modes was calculated for *λ* = 532 nm and *T* = 298 K.

wavenumber, cm⁻¹	type	IR		Raman		participating ions
		active/ inactive	intensity, a.u.	active/ inactive	intensity, relative units	
42.3	B_{1u}	A	35.28	I		Eu, Sc^S, Cu^S, Se1, $Se2^S$
53.7	B_{1g}	I	0	A	1000	Eu^S, Cu^S, $Se1^S$,
55.4	B_{2u}	A	18.31	I		Sc^S, Cu^S, $Se1^S$, $Se2^S$
57.3	B_{2g}	I	0	A	87.60	Cu^S, $Se1^W$,$Se2^S$
66.7	A_g	I	0	A	169.13	Eu^S, Cu^S, $Se1^S$, Se2
66.9	A_u	I	0	I		Sc^S, $Se2^S$
74.0	B_{2g}	I	0	A	31.80	Eu^S, Cu^W,$Se2^W$
74.3	B_{1u}	A	34.48	I		Eu^S, Sc^S, Cu, $Se1^S$, $Se2^W$
83.8	B_{1g}	I	0	A	83.90	Eu^S, Cu^S, Se1, $Se2^W$
97.2	B_{2u}	A	44.2	I		Eu^S, Sc, Cu, Se1, Se2
103.3	A_g	I	0	A	122.47	Eu^S, Cu^S, $Se1^W$, Se2
104.6	B_{3u}	A	190.65	I		Eu^S, Sc^W, Cu^S, $Se1^W$,Se2
107.1	B_{1u}	A	87.97	I		Eu, Sc^S, Cu, Se1, $Se2^S$
119.5	B_{3u}	A	109.68	I		Eu, Sc, Cu, $Se2^S$
136.8	B_{3g}	I	0	A	37.97	$Se2^S$
140.7	B_{1g}	I	0	A	2.21	Eu^W, $Se2^S$
151.0	B_{2g}	I	0	A	1.78	Cu^W, $Se1^S$, $Se2^S$

172.7B$_{3u}$	A	12.99I		Sc, Cu^{S}, Se1^{S}, Se2^{W}
178.8A$_{g}$	I	0A	116.72	Se1, Se2
184.9B$_{3u}$	A	1.44I		Sc^{W}, Cu^{W}, Se1, Se2
185.7A$_{u}$	I	0I		Sc^{S}, Se2
187.1A$_{g}$	I	0A	28.63	Cu^{S}, Se1^{S}, Se2
188.3B$_{2u}$	A	1143.54I		Sc^{S}, Se2
194.3B$_{2g}$	I	0A	37.61	Eu^{W}, Cu^{W}, Se1^{S}, Se2
194.8B$_{1u}$	A	474.57I		Sc, Cu^{S}, Se2
205.9A$_{g}$	I	0A	215.74	Cu, Se1, Se2
209.5B$_{1g}$	I	0A	9.34	Cu, Se1^{S}
212.1B$_{2g}$	I	0A	18.56	Cu, Se1, Se2
212.8B$_{2u}$	A	129.89I		Cu, Se1^{S}
216.2B$_{1u}$	A	601.66I		Sc^{S}, Se1^{S}
258.7B$_{1u}$	A	67.47I		Sc^{S}, Se2^{W}
274.6B$_{3u}$	A	390.67I		Sc^{S}, Cu^{W}, Se1^{W}, Se2^{W}
286.7B$_{3u}$	A	36.18I		Sc^{S}, Se2^{W}

Note: Superscripts "S" and "W" in the last column denote strong and weak ion displacements in this mode, respectively. If the displacement is more or equal 0.02 Å, it is denoted as "S"; if the displacement is 0.005–0.01 Å, it is denoted as "W"; if the displacement is <0.005 Å, the ion is omitted from consideration.

Table S5. Wavenumbers in cm^{-1} and types of the phonon modes at the Γ-point for $EuScCuSe_3$ in the *Pnma* structure. The intensity of the Raman modes was calculated for λ = 532 nm and T = 298 K.

wavenumber, cm^{-1}	type	IR		Raman		participating ions
		active/ inactive	intensity, a.u.	active/ inactive	intensity, relative units	
14.7A$_{g}$		I	0A		25.23	Eu^{S}, Sc^{S}, Se1^{S}, Se2^{S}, Se3^{S}
34.9B$_{1g}$		I	0A		0.06	Eu, Sc^{S}, Cu^{S}, Se1, Se2, Se3^{S}
46.5A$_{u}$		I	0I			Eu, Cu^{S}, Se1^{W}, Se2^{W}, Se3^{S}
47.0A$_{g}$		I	0A		7.28	Eu^{W}, Sc, Cu^{S}, Se1, Se2^{S}, Se3^{W}
48.3B$_{2u}$		A	17.08I			Sc^{S}, Cu^{S}, Se1, Se2, Se3
48.6B$_{3g}$		I	0A		1000	Eu, Cu^{S}, Se3^{S}
48.9B$_{3u}$		A	88.54I			Eu, Sc, Cu^{S}, Se1, Se2^{S}
57.0B$_{1u}$		A	0.15I			Eu^{S}, Cu^{S}, Se1, Se2
61.1B$_{1g}$		I	0A		0.68	Sc^{S}, Cu, Se1^{S}, Se2^{S}, Se3
61.7B$_{1u}$		A	0.62I			Eu^{S}, Cu^{S}, Se1, Se2
62.8B$_{2g}$		I	0A		73.15	Cu^{S}, Se1, Se2, Se3^{W}
65.7A$_{u}$		I	0I			Sc^{S}, Se1^{S}, Se2^{S}
66.1B$_{2g}$		I	0A		0.79	Eu^{S}, Sc, Cu, Se1^{W}, Se2^{W}, Se3
66.9A$_{g}$		I	0A		133.36	Eu, Cu^{S}, Se1^{W}, Se2, Se3
71.7B$_{3g}$		I	0A		7.99	Sc^{S}, Se1^{S}, Se2^{S}
71.9B$_{3u}$		A	8.53I			Eu, Sc, Cu^{S}, Se1, Se2, Se3^{S}
75.2B$_{2g}$		I	0A		17.23	Eu^{S}, Cu^{W}, Se2^{W}
77.6B$_{3u}$		A	39.59I			Eu, Sc, Cu, Se1^{W}, Se2^{W}, Se3^{S}
78.7B$_{3g}$		I	0A		57.51	Eu^{S}, Cu, Se1^{W}, Se2^{W}, Se3^{W}
80.3A$_{u}$		I	0I			Eu^{S}, Cu^{S}, Se3^{W}
81.4A$_{g}$		I	0A		2.8	Eu, Sc^{S}, Cu, Se3^{S}
84.0B$_{1g}$		I	0A		1.02	Eu^{S}, Sc, Cu, Se1^{W},
84.4B$_{2g}$		I	0A		0.41	Eu^{W}, Sc, Cu, Se1, Se2, Se3
93.2B$_{2u}$		A	119.44I			Eu^{S}, Sc, Cu, Se1^{W}, Se2^{W}, Se3^{W}
103.2A$_{g}$		I	0A		94.69	Eu, Cu, Se1^{W}, Se2^{W}, Se3^{W}

$104.9B_{1u}$	A	370.45I		Eu, Sc^{W}, Cu, Se1, Se2, $Se3^{W}$
$107.6B_{3u}$	A	191.32I		Eu^{W}, Sc, Cu^{W}, Se1, Se2, $Se3^{W}$
$111.4B_{3u}$	A	0.05I		Eu, Cu, $Se1^{W}$, $Se2^{W}$
$112.7A_{g}$	I	0A	1.01	Eu^{W}, Sc, Se1, Se2, $Se3^{W}$
$119.1B_{2g}$	I	0A	0.45	Eu^{W}, Sc, Cu, Se1, Se2
$119.3B_{1u}$	A	229.2I		Eu^{W}, Sc, Cu, Se1, Se2
$126.1B_{2u}$	A	0.65I		$Se1^{S}$, $Se2^{S}$
$129.9B_{1g}$	I	0A	33.52	$Se1^{S}$, $Se2^{S}$
$130.0A_{u}$	I	0I		Se1, Se2
$134.1B_{3g}$	I	0A	1.16	Eu^{W}, Se1, Se2
$149.9B_{1u}$	A	0.16I		Cu^{W}, Se1, Se2, Se3
$151.2B_{2g}$	I	0A	1.66	Cu^{W}, Se1, Se2, Se3
$172.0B_{1u}$	A	22.03I		Sc, Cu, $Se1^{W}$, Se3
$175.4B_{2g}$	I	0A	0.89	Sc, Cu, $Se1^{W}$, $Se2^{W}$, Se3
$178.8A_{g}$	I	0A	94.17	Se1, Se2, Se3
$182.2B_{1u}$	A	4.02I		Cu^{W}, Se1, Se3
$184.6A_{u}$	I	0I		Sc^{S}, $Se1^{W}$, $Se2^{W}$
$185.7B_{3g}$	I	0A	0.25	Sc^{S}, $Se1^{W}$, $Se2^{W}$
$185.7B_{3u}$	A	261.78I		Sc^{W}, Cu, Se1, Se3
$186.3A_{g}$	I	0A	19.72	Sc^{W}, Cu, Se1, $Se2^{W}$, Se3
$187.1B_{2u}$	A	2231.26I		Sc^{S}, $Se1^{W}$, $Se2^{W}$
$187.8B_{1g}$	I	0A	0.1	Sc^{S}, $Se1^{W}$, $Se2^{W}$
$187.8B_{2g}$	I	0A	4.39	Sc^{W}, Cu^{W}, Se1, $Se2^{W}$, Se3
$188.7B_{3u}$	A	32.53I		Se1, Se2, $Se3^{W}$
$189.7B_{1u}$	A	0.32I		Sc^{W}, Cu^{W}, $Se1^{W}$, Se2, Se3
$196.7B_{2g}$	I	0A	17.99	Cu^{W}, $Se1^{W}$, Se2, Se3
$196.9A_{g}$	I	0A	50.86	Sc, Cu, Se1, $Se2^{W}$, $Se3^{W}$
$199.2B_{3u}$	A	776.9I		Sc, Cu, $Se1^{W}$, $Se2^{W}$, $Se3^{W}$
$207.6A_{u}$	I	0I		Cu, Se3
$207.6B_{3g}$	I	0A	5.99	Cu, Se3
$210.2A_{g}$	I	0A	106.79	Sc, Cu, $Se1^{W}$, Se2, $Se3^{W}$
$210.5B_{1u}$	A	0.36I		Cu, Se1, Se2, Se3
$211.0B_{2u}$	A	299.08I		Cu, Se3
$211.2B_{1g}$	I	0A	0.57	Cu, Se3
$212.1B_{3u}$	A	235.06I		Sc^{W}, Cu^{W}, Se1, Se2, $Se3^{W}$
$214.0B_{3u}$	A	837.3I		Sc, Cu^{W}, $Se2^{W}$, Se3
$215.0A_{g}$	I	0A	20.41	Sc, Cu^{W}, $Se2^{W}$, Se3
$215.4B_{2g}$	I	0A	17.73	Cu, Se1, Se2, $Se3^{W}$
$258.2B_{3u}$	A	129.34I		Sc^{S}, $Se2^{W}$,
$272.1B_{2g}$	I	0A	0.62	Sc^{S}
$272.6B_{1u}$	A	714.67I		Sc^{S}, Cu^{W}, $Se2^{W}$
$278.5A_{g}$	I	0A	2.9	Sc^{S}, $Se1^{W}$, $Se2^{W}$
$284.5B_{1u}$	A	140.62I		Sc^{S}, $Se1^{W}$, $Se2^{W}$
$309.3B_{2g}$	I	0A	0.05	Sc^{S}, $Se1^{W}$, $Se2^{W}$

Note: Superscripts "S" and "W" in the last column denote strong and weak ion displacements in this mode, respectively. If the displacement is more or equal 0.02 Å, it is denoted as "S"; if the displacement is 0.005–0.01 Å, it is denoted as "W"; if the displacement is <0.005 Å, the ion is omitted from consideration.
